# Educating nursing students for cultural competence in emergencies: a randomized controlled trial

**DOI:** 10.1186/s12912-021-00704-1

**Published:** 2021-09-29

**Authors:** Yosef Kula, Odeya Cohen, Neta Clempert, Orli Grinstein-Cohen, Ortal Slobodin

**Affiliations:** 1 School of Public Health, Faculty of Health Sciences, Ben-Gurion University of the Negev, Beer-Sheva, Israel; 2 Department of Nursing, Recanati School for Community Health Professions, Faculty of Health Sciences, Ben-Gurion University of the Negev, Beer-Sheva, Israel; 3 Department of Middle East Studies, Ben-Gurion Universityof the Negev, Beer-Sheva, Israel; 4 Department of Education, Ben-Gurion University of the Negev, 84105 Beer-Sheva, Israel

**Keywords:** Cultural-competence, Online education, Emergency preparedness, Nursing, RCT

## Abstract

**Background:**

Racial and ethnic minorities suffer significantly more than others in the wake of disasters. Despite the growing recognition of the importance of culturally competent health services, systematic cultural competence training in the medical education system is still scarce, especially in the field of emergency. The current study aimed to examine the effectiveness of an online culturally informed intervention for increasing cultural competence in emergencies among nursing students.

**Methods:**

A randomized controlled trial was used to test the intervention effectiveness in increasing nursing students’ cultural competence in four domains: attitudes, knowledge, skills, and encounters. The study included 72 undergraduate nursing students recruited from two academic institutes. Participants were randomized (1:1 ratio) to an intervention (*n* = 34) and control group (*n* = 38). The study adheres to the Consolidated Standards of Reporting Trials (CONSORT). Data analysis was based on multivariate analysis of variance with repeated measures, followed by post hoc analyses with Bonferroni correction for multiple comparisons.

**Results:**

Results showed that the intervention was effective in increasing the participants’ culturally competent knowledge. The effect of the intervention on the skills domain approached significance. No group differences were identified in the attitudes and the encounters domains.

**Conclusions:**

An online culturally informed intervention, incorporated in the curriculum, was effective in enhancing the cognitive aspect of cultural competence (especially at the basic knowledge and understanding levels), but not other domains. Our results encourage the development of future intervention programs that are based on a deep understanding of local values, needs, and preferences.

## Background

Natural and human-made disasters are priority public health concerns that are associated with adverse physical and mental impact on individuals and communities. While all population members are affected by disasters, research suggests that racial and ethnic minorities are more vulnerable than others to the physical, psychological, and economic effects of disasters [1]. An inclusive approach to disaster and emergency preparedness, response, and recovery activities requires that culturally and linguistically diverse populations are not overlooked [2]. Yet, systematic training in cultural competence is still missing from the medical education curriculum [3], especially in the field of emergency.

The current study examined the effectiveness of a culturally informed online education program in increasing cultural competence during emergencies among nursing students. The program incorporates global knowledge of emergencies with local understating of cultural norms, values, and practices. It may assist in increasing nurses’ cultural competence in different settings.

### Nurses as leaders in emergency preparedness and response

The effectiveness of the healthcare system’s response to a public health emergency or disaster is largely dependent on the surge capacity of the nurse workforce [4]. In many places around the globe, nurses represent the largest segment of the healthcare workforce [5]. Nurses hold rich population-based knowledge, skills, and expertise, engage with diverse professional and community settings, and intensively collaborate with a broad range of healthcare professionals. Therefore, they have a far-reaching influence on health system leaders, individuals, and families when it comes to disaster preparedness, response, and recovery [6].

Large scale local and global disasters and emergencies, such as natural disasters, pandemics, and forced migration have provided opportunities for learning about deployment and involvement of the nursing workforce [7, 8]. Lessons learned from previous events show that preparing for mass casualty events must include consideration of a broad range of planning, educational, and technological challenges [4]. Recognizing the substantive contribution of the nursing workforce in disasters, nurse leaders around the world have identified disaster nursing education and training as a vital need. For example, the American Association of Colleges of Nursing [9] requires that disaster education would be part of their essentials of baccalaureate education). Recently, national nursing education experts, including the Veterans Emergency Management Evaluation Center, Office of Public Health, Veterans Health Administration, U.S. Department of Veterans Affairs, the VA Office of Nursing Services and VHA Office of Emergency Management, have developed consensus recommendations for the advancement of disaster nursing education in the United States. They also initiated a call for action to identify challenges and determine the first action steps in improving the practice of disaster nursing [10]. In 2019, the International Nursing Council (ICN) and the World Health Organization (WHO) jointly proposed a framework for disaster care, offering statements on diagnoses, outcomes and interventions [11]. The ICN Framework of Disaster Nursing Competencies offered eight disaster competencies: preparing and learning, communication, incident management systems, safety and security, assessment, intervention, recovery, and law and ethics [11].

Within all phases of emergency management, disasters highlight and exacerbate social vulnerabilities that require culturally competent care [12, 13]. Therefore, disaster nursing is aimed at protecting not only individuals’ health, livelihoods, and property, but also cultural and environmental assets, such as social cohesion, cultural values, and community resilience. According to the ICN [11], nurses “need to advocate for systems and protocols that protect their ethical obligations as nurses, as well as ensure equity and fairness in disaster medical care planning, while promoting and protecting all human rights, especially those of vulnerable groups such as women, children, the elderly, prisoners, refugees and socially stigmatized groups.”

### Cultural competence in emergency nursing

Cultural competence in healthcare requires a systematic understanding of the cultural and social effects on individuals health-related beliefs and behaviors and on the multiple levels of the healthcare system [14]. The most popular conceptualization of cultural competency, proposed by Sue et al. [15], includes three aspects: (1) awareness of one’s own culturally related biases, attitudes, and values, (2) knowledge about the cultural values and historical background of diverse populations, and (3) specific skills that can be applied to increase effectiveness when working with a diverse clientele.

There are three main reasons why culturally competent nursing may be especially crucial during emergencies. The first reason is the high vulnerability of racial and ethnic minority groups to the physical, mental, and economic effects of disasters [16]. The increased vulnerability of ethnic minority throughout the continuum of disaster phase has been attributed to multiple cultural, social, and financial factors, including the level of language proficiency, limited acculturation level, migration background, lower socioeconomic status, disparities in healthcare, reduced access to information, community isolation, and distrust in healthcare systems [17, 18]. For example, recent reports of the COVID-19 outbreak showed that ethnic and racial minorities were at higher risk for severe morbidity, complications, and death from the virus. This vulnerability was attributed to multiple health, social and economic factors, including urban density, pre-existing medical conditions, lack of information about the disease, and insufficient preparedness efforts [19]. Also, the high interconnectedness of family and community members in collectivist cultures entails that the effects of disasters may impact a wide circle of individuals beyond the direct victims [20]. The lifestyles and behaviors of collectivist countries, such as conformity and tradition, which are a source of physical and social resilience [21], may also pose a threat in times of pandemic outbreaks [22, 23].

The second reason why cultural competency is so important during emergencies is related to the key role that cultural values and traditions play in community resilience. Disaster has been defined as an event in which the social structure is disrupted and prevents the fulfillment of the society’s essential functions [24, 25]. Disasters may also create or exacerbate tensions between racial and ethnic groups, increasing discrimination and racism and putting communities of severe social and economic adversities [26]. Notably, some human-made disasters, such as war, terror, or violence, are often directed towards communities with limited resources that have already experienced a severe disruption to their social fabric due to displacement, loss, trauma, and distrust [27]. Therefore, the strengthening of community reliance and cultural identity is a crucial intervention goal [28]. Finally, cultural competency is essential because crisis interventions require an immediate development of trust between people or organizations [29]. Providing a respectful, empathic, and tolerant professional attitude might be particularly challenging in emergencies because healthcare providers are expected to work under extreme levels of stress, often in non-familiar geographical and socio-cultural contexts.

### The current study

In recent decades, a growing number of educational interventions have been developed to increase nurses’ cultural competence [30–32] .However, there is currently a lack of evidence from rigorous evaluations (e.g., Randomized Controlled Trials; RCT) on the effectiveness of these interventions. Importantly, the influence of culturally competent intervention within disasters and emergency management has not been systematically studied [21]. There is also a lack of specific cultural competency knowledge in the emergency management scholarship of learning and teaching literature [16]. Such lack of emergency- specific cultural education, coupled with the increasing diversity of the patient population [13], underscore the critical need of for cultural competence education and training in medical higher education [33–35]. Theoretical and practical cultural knowledge and skills will help nurses to link cultural competences with evidence-based practice, promote empathic and respectful attitudes, and reduce racial / ethnic biases and stereotypes [4].

Realizing the key role of nurses in emergency and the need to improve cultural competence in early stages of nurses’ education, the aim of the current study was to examine the effectiveness of an online culturally informed intervention in increasing emergency cultural competence in nursing students, using a rigorous study design (RCT). The current intervention incorporates global knowledge of emergencies with local understating of cultural norms, values, and practices.

### The development of a culturally informed intervention

The theoretical framework of the intervention program integrates models of culturally- sensitive mental health interventions [36, 37] with core concepts of cultural competence, such as abilities, knowledge, and skills as set by the International Association of Emergency Managers’ Code of Ethics and Professional Conduct [38]. The research model proposed by Jordans and his colleagues [36] considers both universal and local knowledge of mental health issues. Therefore, it can be easily applied to various emergency contexts. According to the model, developing set culturally competent interventions mandates a preliminary qualitative phase to establish a systematic understanding of the community’s needs and preferences and determine tentative intervention aims. Guided by this view, the described intervention was based on qualitative data collected through semi-structured interviews with ten key- informants in the fields of emergency and cultural competence healthcare. Informants were academic scholars (*n* = 4), military medical professionals (*n* = 2), community physicians (*n* = 3), and the head of one of the largest community emergency response teams. Most of them (70%) were affiliated with one of the ethnic minority groups in Israel (Ethiopian, Muslim-Arabs, Bedouin Arabs, Druze, former-Soviet Union immigrants, and Ultra-orthodox Jews). The analysis was guided by a structured process [39], using the three core concepts of cultural competency [15]: attitudes, knowledge, and skills as a specific theoretical framework. Additionally, the program drew from the education literature, which states that cultural competency is learned through interactive dialogue and reflection exercises [40] and is based on the expertise of staff and faculty [41]. Therefore, the program included short, recorded lectures of academic experts and self-monitoring exercises. Recorded segments of interview data were incorporated as well.

The course syllabus follows the model of cultural competence [42, 43] and reflects the core concepts of cultural competence as set by set by the International Association of Emergency Managers’ Code [38]. The model consists of three aspects of development. The first aspect is concerned with attitudes or awareness towards culture, race, and ethnicity. This aspect includes self-reflection of our personal beliefs, values, and cultural history and how they influence our own and our patients’ lives. The second aspect is concerned with the cultural knowledge of diverse populations. This aspect involves a high motivation to learn about diverse cultures and their health-related beliefs, values, and practices. The third aspect, “skills,” refers to the ability to use cultural knowledge in real-life situations [44].

## Methods

The study is a randomized controlled trial (RCT) designed to assess the effectiveness of a cultural competence intervention for nursing students. The study adheres to the Consolidated Standards of Reporting Trials [CONSORT [45]]. Data was collected between October 2019 and January 2020. For full details about the study protocol, please see Slobodin et al. [46].

### Participants

The initial sample included 186 nursing students recruited from two academic institutes in Israel. Students were offered participation by the course lecturer, who was not part of the research team. A research assistant was present throughout the online course to assist students and address questions. Eligible participants were nursing students who studied in their second academic year at least and completed pre-and post-intervention assessments. Participants that completed less than 80% of the questionnaire were excluded.

**Fig. 1:**
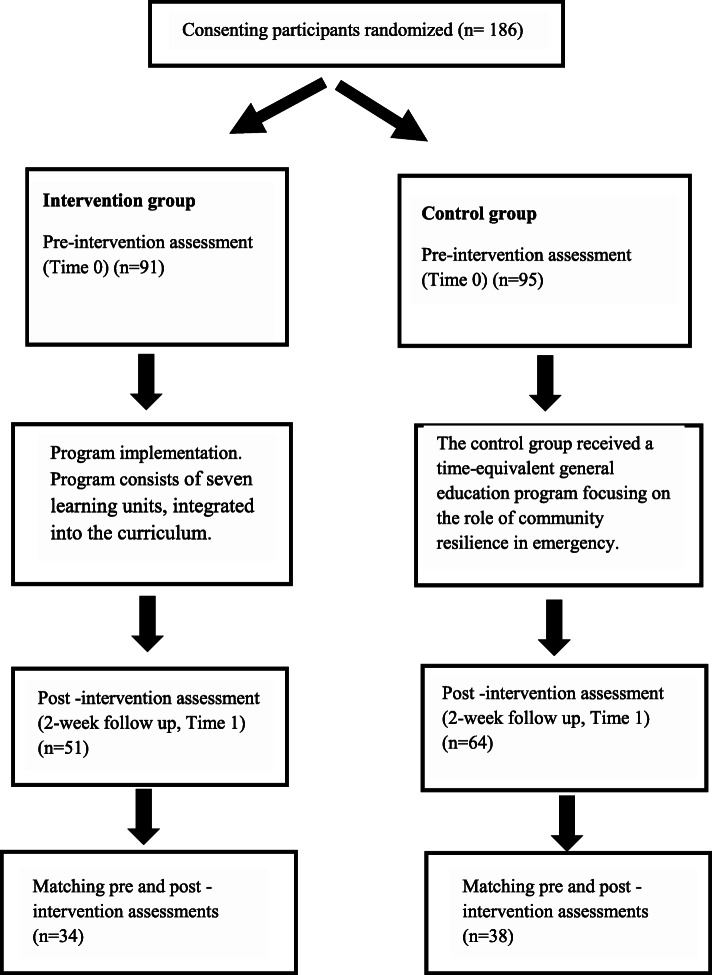
A flow chart of the study methodology and processes

All students signed an informed consent, approved by the IRB, that explains the study’s aims and procedures and emphasizes their voluntary participation and the right to withdraw at any point without consequences. After signing informed consent, the 186 participants were randomized (1:1 ratio) to an intervention and control group. The intervention group was assigned to the cultural competence program. The control group was assigned to a non-intervention condition, an equivalent program addressing general guidelines for clinician-patient communication. Randomization was performed using computer-generated block randomization by an independent researcher. The principal investigators and data analysts were blinded to the group allocations of the participants. Of the 186 randomized participants, 91 participants in the intervention group and 95 in the control group completed the pre-intervention assessment (T0). A total of 115 participants completed the two-week post-intervention assessment (T1), 51 in the intervention group (56%), and 64 (67%) in the control group. Of them, 43 did not provide a consistent personal code that allowed a reliable matching between pre and post assessments. Therefore, they were excluded from the analysis. After pairing pre-and post-intervention assessments, 72 participants were left and included in the final analysis; 34 in the intervention group and 38 in the control group (see Fig. 1 for a flow chart of the study’s methodology).

### Procedure

The course was incorporated into existing academic courses and was delivered as a distant learning program. The cultural competence intervention program consisted of two 60- min sessions, integrated into the curriculum [47, 48]. The control program was an equivalent online program of two 60-min sessions.

Table 1 presents the outline of the cultural competence intervention program and the control program. The cultural competence intervention consisted of seven educational units; (a) Definitions of emergencies (b) The unique challenges facing health services during emergencies c) Introduction to cultural competency (d) Culturally competent attitudes (e) Culturally competent knowledge (f) Culturally competent skills (g Summary.

**Table 1:** **T**he outline of the online cultural competence intervention

	The cultural competence intervention group		Control group	
	Unit	Content	Unit	Content
1	Definitions of emergencies	Different definitions of emergency and their applications. The social, economic and health risks in emergency.	Identical to the intervention group	
2	The unique challenges facing health services during emergencies	The centrality of healthcare services and providers in times of crisis.	Identical to the intervention group	
		Healthcare services play a key role in the response plan, depending on the type of emergency. The importance of healthcare services for individuals and societies’ recovery is far beyond the provision of medical care.		
		Healthcare providers themselves face emotional and professional burdens because of the extreme stress.		
3	Introduction to cultural competency	Defining cultural competence and culturally competent healthcare professionals.	Introduction to community resilience	Community resilience as a multidimensional concept. The shift of the concept from to the field of exact sciences to the field of disaster risk reduction. Definitions of community resilience. The role of community resilience during routine.
		The reasons why ethnic, cultural, and racial minority groups are at high risk to be affected by disasters. Optimal care in times of crisis requires a rapid establishment of positive relationships and trust with patients. To address the needs of diverse population, healthcare providers should develop cultural competence.		
4	Culturally competent attitudes	Recognizing how cultural differences in symptoms manifestation and help-seeking behavior may affect treatment.	The impact of emergency on community structure and cohesion	The reasons why emergencies threaten community cohesion and structure. Emergency has a wide range of impacts on the community networks and structure; communities in these situations react in a rapid re-structure for providing their members’ optimal response. The effects of community’s rapid re-structure on its members and the its narrative.
		Recognizing the effect of one’s own cultural background on personal and professional worldview.		
5	Culturally competent knowledge	Understanding culturally related variation in symptoms manifestation, including mental health problems during routine and emergencies.	The importance of community resilience in times of emergency	The aspects of community resilience, continuity of services, and the importance of critical infrastructures. Interfaces between infrastructures and human capacities.
		Identifying challenges in communication due to language and cultural barriers in times of crisis.		
		Understanding how cultural norms and values (e.g., religious faith) may affect the ways individuals interpret and cope with crisis situations.		
6	Culturally competent skills	Culturally competent medical examination and interview.	Building community resilience in times of crisis	The resilience approach as a pathway between routine and emergency periods. The role of healthcare services in the capacity building of communities. Different strategies to enhance community resilience (e.g., mapping needs and resources; creating two-sides communications between community members and leadership)
		The pros and cons of using an interpreter.		
		The importance of working with key figures within the affected community, including possible pitfalls, especially during emergencies.		
7	Summary		Summary	

Incorporating the control program into the existing academic curricula required that the program provides relevant, educational content. As such, the first two units in the control program units were identical to those of the intervention program. The control program covered the following topics: (a) Definitions of emergencies (b) The unique challenges facing health services during emergencies (c) Introduction to community resilience (d) The impact of emergency on community structure and cohesion (e) The importance of community resilience in times of emergency (f) Building community resilience in emergency (g) Summary. The control program did not include any references to culture or cultural-competent care.

### Measurements

*Background variables*- For each student, age, gender, academic program, academic year, and immigrant status were recorded.

*Cultural Competency-* To evaluate the pre-and post-intervention cultural competency of students, we used a modified version of the Clinical Cultural Competency Questionnaire (CCCQ) [49]. The original questionnaire was developed to assess physicians’ provision of culturally competent healthcare to diverse patient populations. The questionnaire includes 63 items addressing four domains of cultural competence: attitudes (self -assessment of one’s cultural values, beliefs, and behaviors, e.g., “Awareness of own racial, ethnic, or cultural stereotypes”), knowledge (search for knowledge about different cultural groups, e.g., “Knowledge on health disparities”), skills (the ability to accurately and thoroughly assess cultural need, e.g., “Providing culturally competent clinical preventive services”), and encounters (active engagement indirect interaction with different cultural groups, e.g., “Caring for patients from culturally diverse backgrounds”). The CCCQ was used in various cultural settings to measure the effectiveness of cultural competence training programs and demonstrated high reliability [50]. In the current study, the Cronbach’s alphas (at T0 assessment) were .76 for the attitudes domain, .79 for the knowledge domain,.88 for the skills domain, and .89 for the encounters domain. Cronbach’s alpha for the total scale was excellent (.91).

### Data analyses

Based on previous findings regarding multicultural education ([51], for meta-analysis), the expected effect size of the educational program was d = 0.49. Taking this estimation into account, the study required sample size of 35 pairs to achieve a power of 80% and a level of significance of 5%. To examine group differences in changes of CCCQ, we performed a two-way repeated-measures analysis of variance (ANOVA). CCCQ domains (attitudes, knowledge, skills, and encounters) and time (pre vs. post-intervention) were the within-subject factors, and group (intervention vs. control) was the between-subject factor. All multivariate analyses were followed by posthoc analyses with Bonferroni corrections for multiple comparisons. The Statistical Package for the Social Sciences (SPSS) version 26 [52] was used for data analyses.

## Results

### Baseline between-group comparisons

T-tests for independent samples and Chi-square tests were used to compare the two groups on baseline demographic characteristics. The demographic and background characteristics of the two study groups are presented in Table 2. As seen in the table, no group differences were found in age, gender, academic year, and migration background. Group differences in baseline levels of cultural competence were examined using Univariate analysis of variance (ANOVA). No group differences were identified (F (4,66) =2.16, *p* = 0.08).

**Table 2:** Background variables of the two study groups

	Intervention group (*n* = 34)		Control group (*n* = 38)		Difference
Age (M, S.D)	33.71	85.83	32.42	9.88	t (70) =0.58, *p* = .57
Academic year (M, S.D)	2	0.65	2	0.86	t (64.94) =0.00, *p* = 1.00
Gender					
Male	4	11.8%	8	21.1%	
Female	30	88.2%	30	79.9%	χ^2^ (1) =1.12, *p* = .29
Immigrant status	12	35.3%	9	24.3%	χ^2^ (1) =1.02, *p* = .31
Base-line CCCQ scores	M	S.D	M	S.D	
Attitudes	3.32	0.84	3.46	0.98	F (4,66) =2.16, *p* = 0.08
Knowledge	3.04	0.68	2.95	0.61	
Skills	3.11	0.83	3.47	0.73	
Encounters	3.58	0.74	3.53	0.76	

### Multivariate analysis of variance with repeated measures

To examine group differences in the four CCCQ domains, we performed two-way repeated-measures ANOVA (Table 3). Analyses revealed a main effect of domain, Wilks’ Lamda value = 0.53, *F* (3,67) = 20.21, *p* < .001, and for time, Wilks’ Lamda value = 0.612, *F* (1,69) = 43.73, *p < .001*. Post hoc analysis of the domain effect with Bonferroni correction for multiple comparisons indicated that regardless of their group affiliation (intervention vs. control) and of assessment time (pre-intervention vs. post-intervention), participants rated their culturally competent knowledge (M = 3.23, S.D. =0.06) lower than their culturally competent skills (M = 3.53, S.D. =0.08), and comfort (M = 3.72, S.D. =0.07). In addition, participants rated their culturally competent skills lower than their comfort. No difference was found between participants’ rates of their culturally competent attitudes (M = 3.50, S.D. =0.08) and the other three CCQ domains.

**Table 3:** Group differences at base-line and post-intervention CCCQ scores

CCCQ domain	Intervention group (*n* = 34)				Control group (*n* = 38)				Between-group differences in post-intervention CCCQ^a^
	Base-line		Post-intervention		Base-line		Post-intervention		
	Mean	S.D	Mean	S.D	Mean	S.D	Mean	S.D	
Attitudes	3.32	0.84	3.58	0.78	3.46	0.98	3.58	0.76	F (1,69) < 1
Knowledge	3.04	0.68	3.66	0.65	2.95	0.61	3.34	0.51	F (1,69) =3.05, *p* = 0.02
Skills	3.11	0.83	3.84	0.77	3.47	0.73	3.71	0.69	F (1,68) =3.33, *p* = 0.07
Encounters	3.58	0.74	4.00	0.65	3.53	0.76	3.80	0.65	F (1,69) =1.77, *p* = 0.19

^a^With base-line CCCQ levels as covariates

Post hoc analysis of the time effect with Bonferroni correction for multiple comparisons showed that regardless of their group affiliation (intervention vs. control) and of CCCQ domain, participants rated their cultural competence level higher in the post-intervention assessment (M = 3.69, S.D. = 0.05) than in the pre-intervention assessment (M = 3.31, S.D. = 0.06).

The two-way interaction between group and time was significant, Wilks’ Lamda value = 0.934, *F* (1,69) = 4.84, *p* = 0.03 as well as the two-way interaction between group and domain, Wilks’ Lamda value = 0.883, *F* (3,66) = 2.96, *p* = 0.04. The interaction between time and domain was also significant, Wilks’ Lamda value = 0.882, *F* (3,67) = 2.98, *p* = 0.04. The three-way interaction group * time * domain was not significant, Wilks’ Lamda value = 0.949, *F* (3,67) = 1.45, *p* = 0.23. The effect of group (between-subject factor) was not significant, F (1,69) < 1.

**Fig. 2:**
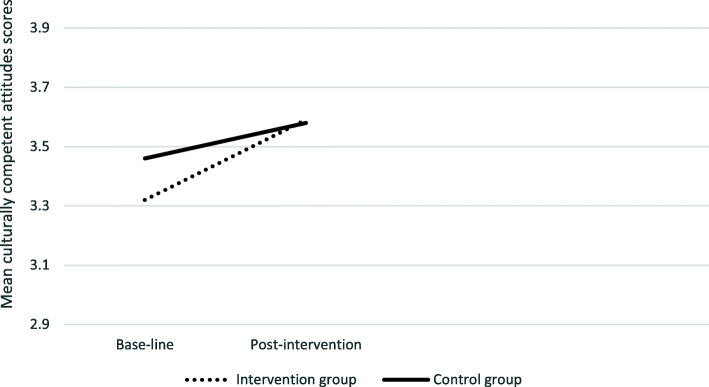
Group differences in the culturally competent attitudes

**Fig. 3:**
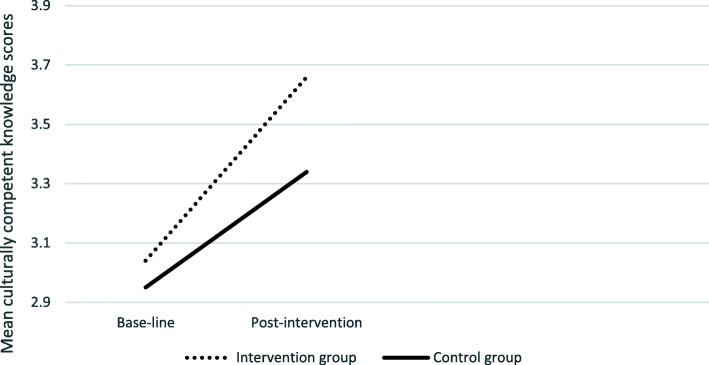
Group differences in the culturally competent knowledge

**Fig. 4:**
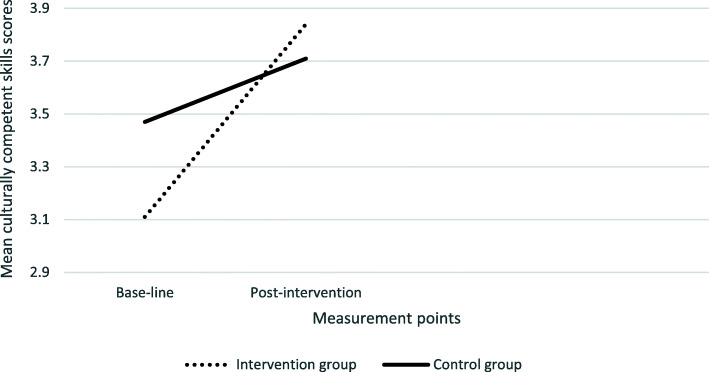
Group differences in the culturally competent skills

Inspection of the interaction plots (see Figs. 2, 3, 4. 5), confirmed by univariate analysis for each CCCQ domain (with the baseline score of each domain as a covariate), revealed significant pre−/post- intervention improvement in the knowledge domain; F (1,69) =3.05, *p* = .024 and a marginal effect on the skills domain; F (1,68) = 3.33, *p* = 0.07. No group differences were identified in the attitudes F (1,69) < 1 and the encounters domain; F (1,69) = 1.77, *p* = .19.

**Fig. 5:**
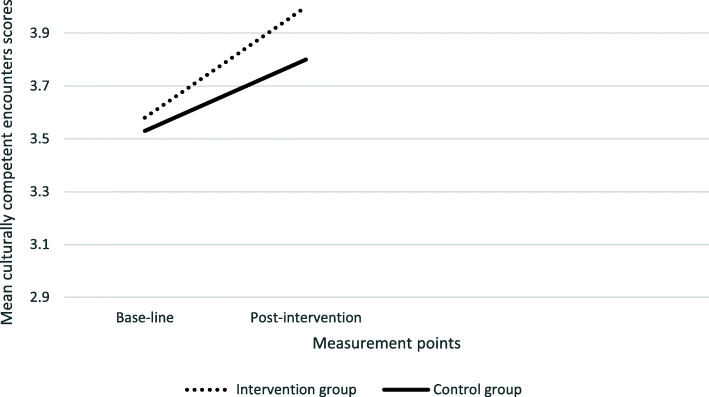
Group differences in the culturally competent encounters

## Discussion

As the role of nurses in emergency management field continues to grow and mature, the notion of culturally competent care is being incorporated into professional standards and medical education. To date, research examining cultural competence training has overlooked the context of emergency and suffered from methodological constraints, such as weak study designs (e.g., lack of RCTs), low or no reporting of consent rates, and non-validated measurement instruments, limiting rigorous evaluations on the effectiveness of interventions [14, 53]. The current study examined the effectiveness of an online intervention in increasing nursing students’ emergency cultural competence, using a RCT design.

Overall, our results supported the use of an online program incorporated in the curriculum for increasing students’ cultural competence. Specifically, participants in the intervention group were more likely to report increased knowledge of socio-cultural characteristics, health disparities, and health risks experienced by particular racial and ethnic groups, as well as of alternative healing traditions and methods [54]. The effect of the intervention in increasing participants’ ability to translate this knowledge into their daily practice (skills) approached significance. However, participants did not present increased confidence (encounters) in their ability to empower patients from diverse backgrounds or increased awareness of their own cultural background, stereotypes, or biases. These findings are consistent with previous studies [55–57] and systematic reviews [58, 59] that examined the efficacy of cultural competence in non-emergency contexts, showing that cultural competence training is especially effective in facilitating cultural-competence knowledge. For instance, a systematic review of 16 studies by Jongen et al. [59] found that cultural competence training improved knowledge in 9 of 16 studies, skills in 7 of 16, attitudes/beliefs in 5 of 16 studies, and confidence in 5 studies.

Several explanations may be proposed but warrant further exploration. First, focused intervention programs incorporated in the curriculum context are more likely to enhance the cognitive aspect of cultural competence (especially at the basic knowledge and understanding levels) than other domains. From an education perspective, culturally- diverse knowledge is easier to learn and teach than practical skills or attitudes (affective learning domain) [60]. Second, changes in culturally competent knowledge are easier to measure than changes in other domains [61]. The gains in culturally competent knowledge may also be attributed to the course delivery mode [58]. While different cultural competence training was delivered by professional trainers [55], sometimes from diverse backgrounds [56], our intervention was provided in two online sessions. Such delivery mode, especially in academic settings, may focus on the acquisition of facts rather than encouraging affective engagement or self-reflection. It is possible that longer courses that include active participation (e.g., students’ presentations, classroom discussions, simulations) would increase the practical and affective domains of cultural competence.

A further explanation for our findings is related to the considerable overlap between the control and the intervention programs in both content and structure. Given that the control program was incorporated within the curriculum, it was necessary to develop a course that consists of relevant educational content. The control program therefore addressed the pivotal role of healthcare services during emergencies and emphasized the importance of social communication and networks. While not explicitly addressing culturally related topics, this program may encourage a patient-centered approach and promote sensitive and empathic attitudes among students. Indeed, our results showed that, compared to the pre-intervention assessment, both the intervention and the control groups showed an increase in their CCCQ scores. Such similarity between the intervention and control programs may explain why the culturally related knowledge was the most prominent gain from the intervention. Because most cultural competency training studies did not include a comparison group or used a non-intervention control group ([3], for review), there is a need for a systematic investigation of what could be considered a “comparison condition.” For example, Genao et al. [62], who examined a cultural competence curriculum for third-year medical students, presented a control program that included lectures on clinical preventive medicine, alternative medicine, and domestic violence, taught by faculty with expertise in those areas. This program, however, did not necessarily encourage a patient-centered approach, and therefore might be more distinguished from the intervention group than in our study.

The limited differences between the intervention and control group might also be attributed to the demographic nature of our sample, which consisted of a high proportion of immigrants (30%). Previous studies suggested that compared to white therapists, cultural and linguistically diverse professionals were more likely to be involved with ethnic minority communities, to use a cultural framework in their clinical practice, and to perceive their agencies as culturally sensitive [63]. Ethnic minority healthcare professionals often share patient’s experiences of racism and prejudice [64], motivating them to provide more culturally competent care. It is possible that healthcare students of immigrant backgrounds were already aware of the importance of culturally- competent care and familiar with the concepts of cultural competence [65]. Therefore, the only effect of the intervention program was evident in the practical skills domain, where training was necessary.

Our study had several notable strengths. First, the study used a theory-based intervention that integrates universal as well as local, cultural-specific understating of crisis responses and resilience. Such integrated approach is a novel application in medical education research that may guide investigators, practitioners and educators who are interested in tailoring culturally competent interventions in various cultural and emergency settings. Second, the current study is built on the model of methodological excellence in educational studies [66] that advocates for the use of blind RCTs with valid instruments and appropriate statistical analyses of subgroups. Thus, we reduced the risks of confounding and selection biases. This research is also one of the few studies that examined the efficacy of cultural competence training programs outside the United States. On the practical level, the described intervention offers a promising strategy of creating, implementing, and evaluating a cultural competence program specifically designed for emergency management in higher education.

The reported findings should be considered under several limitations. First, this study relied on self-report measures of cultural competence and did not include an objective evaluation method, such as health outcomes ([67], for a systematic review) or patient satisfaction ([53], for a systematic review). Self-report measures are also vulnerable to various biases, including social-desirability or response-shift bias, that may confound the intervention effect with bias recalibration [68]. Second, because the post-intervention assessment did not include a follow-up phase, it is difficult to determine whether the intervention’s advantage would be stable over time. Third, because this study was based on healthcare students, our ability to generalize our results to other healthcare populations and setting is currently limited. Finally, due to the high attrition rate, the sample size was limited.

## Conclusions

Immigration and the growth of multicultural societies have highlighted the need for culturally competent care worldwide, especially in times of emergencies. Our results encourage the development of future intervention programs that are based on a deep understanding of local needs and preferences and incorporate ethnographic cultural knowledge. Equally important is the usage of large-scale randomized controlled trials that would evaluate real-life, cultural competence and not only self-report measures. There is also a need to examine the applicability of cultural competence training programs to different emergencies and to adapt their content and structure to the specific needs of the disaster and the patient population.

## Data Availability

The datasets used and analyzed during the current study are available from the corresponding author on reasonable request.

## Abbreviations

ANOVA: Analysis of Variance
CCCQ: Clinical Cultural Competency Questionnaire
CONSORT: Consolidated Standards of Reporting Trials
ICN: International Nursing Council
RCT: Randomized Controlled Trial
WHO: World Health Organization
